# Diagnostic accuracy of circulating blood biomarker families in acute ischemic stroke: a secondary grouped meta-analysis with primary-study verification

**DOI:** 10.3389/fneur.2026.1856224

**Published:** 2026-09-24

**Authors:** Xiao Luo, Longfeng Ran, Yao Wu, Xuan Zhang, Liu Wu, Xin Mu

**Affiliations:** 1 Department of Neurology, Chengdu Integrated TCM & Western Medicine Hospital, Chengdu, Sichuan, China; 2 Qionglai Hospital of Traditional Chinese Medicine, Chengdu, Sichuan, China

**Keywords:** acute ischemic stroke, blood biomarker, cell-free DNA, diagnostic accuracy, HIF-1alpha, non-coding RNA, secondary meta-analysis

## Abstract

**Background:**

Circulating biomarkers may complement clinical assessment and neuroimaging during the early evaluation of suspected acute ischemic stroke (AIS), but the evidence spans biologically and analytically heterogeneous test families. We revised the original analysis as a transparent secondary grouped diagnostic accuracy meta-analysis and verified all contributing primary reports.

**Methods:**

Candidate studies were obtained from two recent systematic reviews of acute-window protein and cell-free nucleic-acid biomarkers, backward citation checking, and a targeted PubMed/MEDLINE update through 31 July 2026. Primary reports were verified against predefined eligibility criteria. Biomarkers were reclassified as extracellular chromatin/cell-free DNA, hypoxia-response, non-coding RNA, or protein/multimarker tests. Where published 2 × 2 tables were unavailable, cells were reconstructed from sensitivity, specificity, and diseased/non-diseased sample sizes using prespecified rounding rules. A bivariate random-effects model was used only when at least four independent cohorts were available; families with fewer cohorts were summarized descriptively. QUADAS-2, comparator-spectrum analyses, one-estimate-per-cohort rules, and rounding-bound sensitivity analyses were added.

**Results:**

Seventeen verified primary reports published from 2003 to 2025 contributed 24 original diagnostic test estimates. The cohort-level primary dataset contained 17 independent cohorts. Extracellular chromatin/cell-free DNA tests (four cohorts) yielded pooled sensitivity 0.712 (95% CI 0.454–0.880), specificity 0.796 (0.699–0.868), LR + 3.50 (2.27–5.40), LR − 0.36 (0.17–0.76), and DOR 9.68 (3.39–27.62). Non-coding RNA tests (10 cohorts) yielded sensitivity 0.866 (0.829–0.896) and specificity 0.922 (0.585–0.990), but the wide specificity and DOR intervals indicated substantial uncertainty. Hypoxia-response and protein/panel evidence comprised only one and two independent cohorts, respectively, and was not formally pooled. Most studies used healthy-control two-gate designs and data-driven thresholds; all diagnostic 2 × 2 tables required reconstruction. Comparator-, rounding-, dependence-, and leave-one-cohort-out analyses preserved the direction of the main findings but demonstrated substantial uncertainty and spectrum effects.

**Conclusion:**

Non-coding RNA and extracellular chromatin/cell-free DNA tests show promising but methodologically fragile diagnostic signals. The evidence is not sufficient for routine clinical implementation because realistic stroke-mimic cohorts are sparse, thresholds are usually data-driven, and reconstructed cells and within-cohort multiple testing remain important limitations. Prospective one-gate, mimic-enriched validation is required.

## 1 Introduction

Acute ischemic stroke (AIS) remains a major cause of death and long-term disability, and benefit from reperfusion therapy depends on rapid recognition, imaging, and treatment (1–5). Neuroimaging remains indispensable, but early diagnosis can be complicated by mild deficits, posterior-circulation presentations, wake-up stroke, and common stroke mimics. A blood test that is rapid, reproducible, and additive to clinical examination could therefore support triage or diagnostic confidence; it should not be viewed as a replacement for computed tomography or magnetic resonance imaging (6–9).

The AIS biomarker literature is heterogeneous in both biology and design. Protein markers reflect neuronal, glial, endothelial, cardiac, or inflammatory injury; extracellular chromatin and cell-free DNA may index tissue injury and immunothrombosis; hypoxia-response markers reflect oxygen-sensing pathways; and non-coding RNAs may integrate tissue-specific transcriptional responses (6–14). Reported diagnostic performance is strongly affected by symptom-to-sampling time, assay platform, threshold selection, and comparator spectrum. Studies that contrast AIS with healthy volunteers often yield more favorable estimates than studies enrolling consecutive patients with stroke-like symptoms, a spectrum effect that limits direct cross-family ranking (6–9).

The original manuscript grouped several miRNA, lncRNA, and circRNA tests under the term ‘NETs-related extended biomarkers’. That label implied a biological relationship not established for many component markers. In this revision, the group was renamed ‘non-coding RNA biomarkers’, while histone, dsDNA, and cfDNA assays were retained in a narrower extracellular chromatin/cell-free DNA group. Hypoxia-response biomarkers and protein/multimarker tests were treated separately. This revision also separates diagnostic evidence from the supplementary prognostic analyses of Cit-H3 and HIF-1alpha.

The present work is explicitly framed as a secondary grouped diagnostic accuracy meta-analysis rather than a *de novo* systematic review. We reconstructed the evidence provenance from two recent systematic reviews, verified the primary reports, and conducted a targeted update search (8, 14). The objectives were to (1) compare diagnostic behavior within biologically coherent test families; (2) identify the extent to which comparator type, sampling window, and multiple tests per cohort affect interpretation; (3) document risk of bias with QUADAS-2; and (4) evaluate the robustness of reconstructed 2 × 2 cells using prespecified sensitivity analyses (15–19).

## 2 Methods

### 2.1 Study design and evidence provenance

This study is a secondary grouped diagnostic accuracy meta-analysis with primary-study verification. The pre-revision dataset had been assembled from earlier evidence-extraction work, but the original screening log and database-level record counts were not retained. We therefore did not retrospectively invent PRISMA record counts. Instead, the revision reconstructed a transparent evidence-provenance pathway using two anchor systematic reviews—one focused on blood protein biomarkers during the acute treatment window and one focused on cell-free nucleic acids—followed by primary-report verification, backward citation checking, and a targeted PubMed/MEDLINE update through 31 July 2026 (8, 14). The review protocol was not prospectively registered; this is reported as a limitation.

### 2.2 Targeted search and source verification

The PubMed/MEDLINE update combined terms for AIS or cerebral infarction, blood/serum/plasma biomarkers, diagnostic accuracy, and the candidate families (cell-free DNA, extracellular DNA, histones, HIF-1alpha, miRNA, lncRNA, circRNA, H-FABP, S100B, and multimarker panels). No language restriction was applied. Full search syntax is provided in Supplementary Table S1. For every included report, bibliographic identifiers, sample sizes, test names, comparator populations, sampling windows, sensitivity, specificity, AUC, and thresholds were checked against the primary article or PubMed record. The revised analysis includes only sources for which a primary report could be identified; the previous placeholder ‘UnknownRef46’ was resolved as Reynolds et al. (31).

### 2.3 Eligibility criteria

Eligible reports enrolled adults with AIS diagnosed by clinical neurological assessment supported by CT or MRI; evaluated a circulating blood, serum, plasma, or peripheral-blood-cell biomarker for AIS diagnosis or differential diagnosis; included a non-AIS comparator group; and reported TP, FP, FN, and TN or sufficient information to reconstruct these cells from sensitivity, specificity, and group denominators. Prospective and retrospective diagnostic cohorts and case–control studies were eligible. Exclusion criteria were animal or in vitro studies, tissue/thrombus-only assays, cerebrospinal-fluid-only tests, studies limited to prognosis without diagnostic accuracy, reports providing AUC without recoverable group-specific accuracy data, duplicate cohorts without an analytically distinct test, and records whose primary source or denominators could not be verified.

### 2.4 Unit of analysis and management of multiple tests per cohort

The original dataset contained multiple tests or subtype estimates from several cohorts. Counting these estimates as independent studies would understate uncertainty. We therefore distinguished primary reports, independent cohorts, and diagnostic test estimates. The main cohort-level analysis retained one prespecified estimate per cohort: the article-designated primary test or validated panel was preferred; an independent replication cohort was preferred over a discovery cohort; and a panel was analyzed separately from its component tests. For Tian et al., diseased subtype counts were combined while the shared control group was counted once. Companion markers were retained in the complete extraction table and used only in arm-level sensitivity analyses.

### 2.5 Biomarker-family classification

Tests were classified using explicit biological and analytical criteria. The extracellular chromatin/cell-free DNA family included circulating histone H3.1, dsDNA, TERT-targeted cfDNA, and beta-globin cfDNA. The hypoxia-response family included HIF-1alpha and the linked miR-210 test from the same cohort. The non-coding RNA family included miRNAs, lncRNAs, and circRNAs irrespective of whether they had a direct NET relationship. The protein/multimarker family included H-FABP, S100B, and protein-panel algorithms. Cit-H3 prognostic associations were not treated as diagnostic test accuracy data and were moved to a supplementary prognostic analysis.

### 2.6 Reconstruction of diagnostic 2 × 2 tables

No included report provided a complete directly extractable 2 × 2 table for the final selected threshold. When sensitivity (Se), specificity (Sp), the number with AIS (N_AIS), and the number without AIS (N_non-AIS) were available, cells were reconstructed as TP = round(Se × N_AIS), FN = N_AIS − TP, TN = round(Sp × N_non-AIS), and FP = N_non-AIS − TN. Reconstructed cells were checked by recomputing sensitivity and specificity. Because excluding reconstructed datasets would leave no analyzable diagnostic cohort, we replaced the requested exclusion analysis with a prespecified rounding-bound sensitivity analysis using floor, nearest-integer, and ceiling rules. The data-origin field is provided for every estimate in Supplementary Table S2, and source identifiers/URLs are provided in Supplementary Table S3.

### 2.7 Risk-of-bias assessment

Risk of bias and applicability were assessed with QUADAS-2 across patient selection, index test, reference standard, and flow/timing domains (17). Patient-selection risk was judged high for selected two-gate case–control designs and unclear when consecutive or random enrollment was not documented. Index-test risk was judged high when diagnostic thresholds were selected from the same ROC dataset rather than prespecified. CT/MRI-supported clinical diagnosis was considered an acceptable reference standard. Study-level judgments and written rationales are reported in Supplementary Table S4, Figure 1 and Supplementary Figure S1.

**Figure 1:**
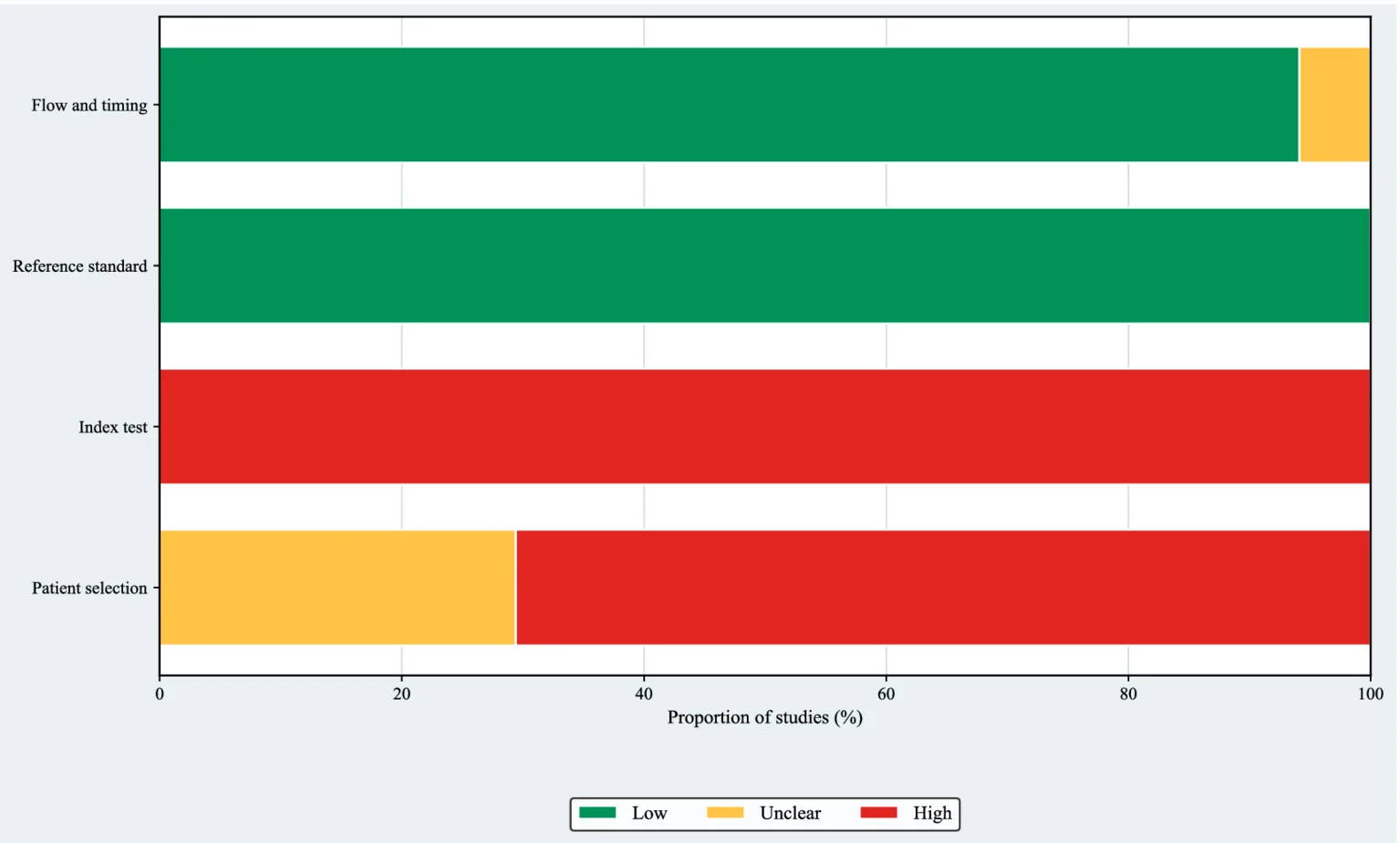
QUADAS-2 risk-of-bias summary across 17 verified primary reports. High index-test risk primarily reflects data-driven or non-prespecified thresholds; high patient-selection risk primarily reflects two-gate healthy-control designs.

### 2.8 Statistical analysis

For families represented by at least four independent cohorts, sensitivity and specificity were jointly synthesized with a bivariate random-effects model on the logit scale (22–25). Summary LR+, LR−, and DOR were derived from the joint summary point. A 0.5 continuity correction was used only for ratio estimates when a cell was zero. Study-level sensitivity and specificity forests and summary ROC displays were prepared. Families with fewer than four independent cohorts were summarized descriptively rather than forced into unstable hierarchical models. To address comparator and timing heterogeneity without overfitting sparse hierarchical models, exploratory subgroup displays summarized sensitivity and specificity separately with univariate random-effects logit models; these subgroup estimates were used for visualization and did not replace the primary family-specific bivariate models. Formal meta-regression was not performed because no covariate had approximately 10 or more independent cohorts. Deeks asymmetry testing was restricted to the non-coding RNA family (10 independent cohorts) and interpreted as exploratory (26). Analyses were aligned with Stata/MP 17.0, MIDAS (SSC S456880, revised 23 June 2026), and metandi as described by Harbord and Whiting (27, 28).

### 2.9 Sensitivity and supplementary analyses

Sensitivity analyses addressed three distinct sources of instability: within-cohort multiplicity, rounding of reconstructed 2 × 2 cells, and single-cohort influence. The primary analysis used one estimate per independent cohort; all eligible test estimates from shared cohorts were retained only for a dependence audit. Reconstruction robustness was evaluated with floor, nearest-integer, and ceiling rules. Influence was examined with leave-one-cohort-out random-effects DOR analyses using the same continuity-correction convention for zero cells. Prognostic odds-ratio estimates for Cit-H3 and HIF-1alpha were analyzed separately with random-effects inverse-variance methods and are reported in the supplement; they do not contribute to the diagnostic accuracy estimates.

## 3 Results

### 3.1 Evidence base and study flow

Seventeen verified primary reports published from 2003 through 2025 contributed 24 original diagnostic test estimates. After applying the one-estimate-per-cohort rule, the primary dataset contained 17 cohort-level estimates: four extracellular chromatin/cfDNA cohorts, one hypoxia-response cohort, 10 non-coding RNA cohorts, and two protein/multimarker cohorts (Figure 2 and Tables 1, 2). Eleven cohorts used healthy controls, three used stroke mimics, two used hemorrhagic-stroke comparators, and one used vascular-risk controls. Because the pre-revision screening log was unavailable, Figure 2 is an evidence-provenance flow rather than a conventional PRISMA diagram with unrecoverable record counts.

**Figure 2:**
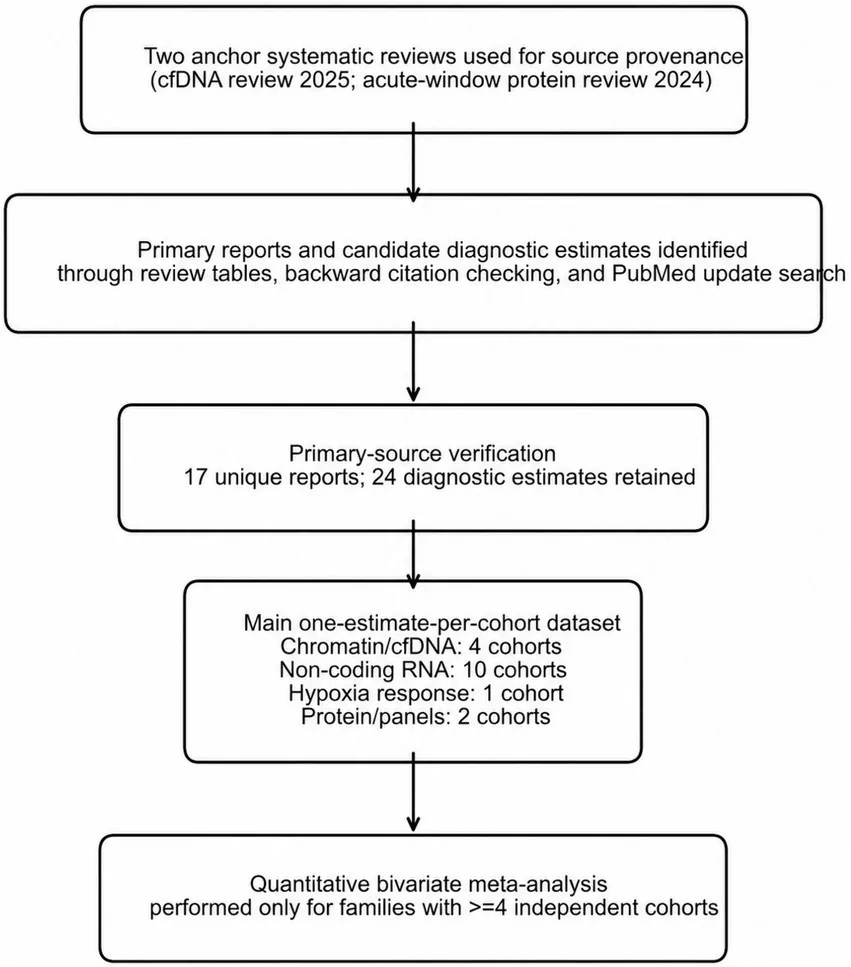
Evidence provenance and primary-study verification flow. Because the original pre-revision database screening log was unavailable, the figure reports the transparent secondary evidence pathway rather than unrecoverable PRISMA record counts.

**Table 1:** Characteristics of the verified cohort-level primary dataset.

Study	Family	Index test	Comparator	Sampling	AIS/non-AIS	Data origin
Rainer (2007) (58)	Extracellular chromatin/cfDNA	cfDNA (beta-globin)	Hemorrhagic stroke	Within 24 h	118/35	Reconstructed from Se/Sp and group sizes
O’Connell (2017) (57)	Extracellular chromatin/cfDNA	cfDNA (TERT)	Stroke mimic	Emergency admission	43/20	Reconstructed from Se/Sp and group sizes
Lim (2020) (62)	Extracellular chromatin/cfDNA	dsDNA	Healthy control	Initial presentation	58/25	Reconstructed from Se/Sp and group sizes
Park (2024) (55)	Extracellular chromatin/cfDNA	Histone H3.1	Healthy control	At presentation	89/20	Reconstructed from Se/Sp and group sizes
Rahmati (2021) (47)	Hypoxia-response	HIF-1alpha	Healthy control	Admission	52/52	Reconstructed from Se/Sp and group sizes
Peng (2015) (34)	Non-coding RNA	let-7e	Healthy control	Acute stage	72/51	Reconstructed from Se/Sp and group sizes
Tian (2016) (35)	Non-coding RNA	miR-16 (combined TACI/LAA)	Healthy control	6 h	13/23	Combined from subtype data with shared controls
Wang (2017) (42)	Non-coding RNA	lncRNA H19	Healthy control	Within 3 h	36/25	Reconstructed from Se/Sp and group sizes
Tiedt (2017) (33)	Non-coding RNA	miR-125a-5p/miR-125b-5p/miR-143-3p panel	Healthy control	On hospital arrival (<24 h)	200/100	Reconstructed from Se/Sp and group sizes
Gui (2019) (40)	Non-coding RNA	let-7e	Healthy control	Within 24 h	28/20	Reconstructed from Se/Sp and group sizes
Zhou (2021) (36)	Non-coding RNA	miR-124	Healthy control	Within 24 h	108/108	Reconstructed from Se/Sp and group sizes
Adly Sadik (2021) (38)	Non-coding RNA	miR-155	Healthy control	Within 24 h	46/50	Reconstructed from Se/Sp and group sizes
Ewida (2021) (41)	Non-coding RNA	lncRNA LINK-A	Hemorrhagic stroke	Within 24 h	50/25	Reconstructed from Se/Sp and group sizes
Liu (2022) (39)	Non-coding RNA	circOGDH	Risk control	Within 24 h	45/32	Reconstructed from Se/Sp and group sizes
Jiang (2025) (29)	Non-coding RNA	hsa_circ_0005729	Healthy control	Admission	144/144	Reconstructed from Se/Sp and group sizes
Reynolds (2003) (31)	Protein/multimarker panels	vWF/MMP-9/S100B/MCP-1/BNGF panel	Stroke mimic	Within 6–12 h	82/214	Reconstructed from primary-source sensitivity/specificity and reported group sizes
Park (2013) (20)	Protein/multimarker panels	H-FABP + S100B panel	Stroke mimic	<24 h	111/127	Reconstructed from Se/Sp and group sizes

**Table 2:** Revised biomarker-family classification and analytical rationale.

Family	Included tests	Classification criterion	Independent cohorts
Extracellular chromatin/cfDNA	Histone H3.1, dsDNA, TERT-cfDNA, beta-globin cfDNA	Direct chromatin/NET-associated or cell-free DNA measures	4
Hypoxia-response	HIF-1alpha; companion miR-210	Oxygen-sensing response measured in the same cohort	1
Non-coding RNA	miRNA, lncRNA, circRNA tests and RNA panels	RNA classifiers without assuming a NET-specific relationship	10
Protein/multimarker panels	H-FABP, S100B, five-protein panel	Single proteins or protein algorithms	2

### 3.2 Study and test characteristics

Blood was collected from presentation to 24 h in most reports. Plasma, serum, whole blood, and PBMC-derived RNA were represented. qRT-PCR predominated in the non-coding RNA family; ELISA or fluorometric/qPCR assays were used for chromatin/cfDNA; and protein studies used single-analyte ELISA or multimarker algorithms. The comparator spectrum differed sharply by family: eight of 10 non-coding RNA cohorts used healthy controls, whereas the two protein/panel cohorts used stroke mimics. This imbalance precluded a clinically valid direct ranking across families (Figure 3 and Supplementary Table S2).

**Figure 3:**
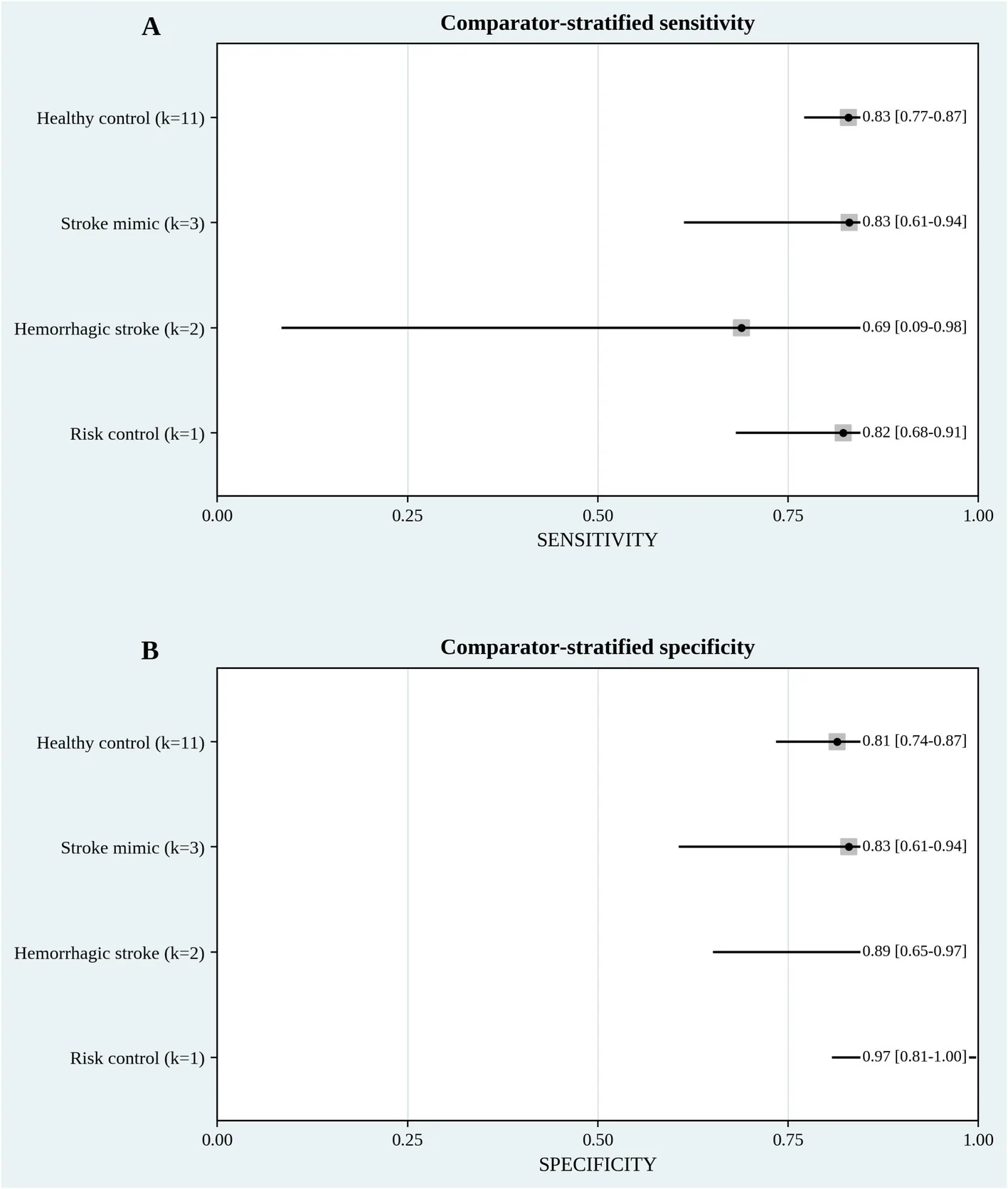
Exploratory comparator-stratified diagnostic performance. Panels **(A,B)** show sensitivity and specificity, respectively, grouped by comparator type. Values were summarized separately with univariate random-effects logit models for visualization and do not replace the primary family-specific bivariate models. The risk-control stratum contains one cohort and is shown descriptively. Comparator strata contain different biomarker families and should not be interpreted as head-to-head test comparisons.

### 3.3 Risk of bias

QUADAS-2 identified patient selection and threshold selection as the principal limitations (Figure 1 and Supplementary Figure S1). Twelve of 17 reports were judged high risk for patient selection, primarily because they used selected healthy-control or two-gate designs; the remaining five were unclear because consecutive enrollment was incompletely reported. All reports were judged high risk for the index-test domain because thresholds were data-driven or not demonstrably prespecified. Reference-standard risk was low in all reports because AIS status was established by clinical assessment supported by CT or MRI. Flow/timing was generally low risk, with one report judged unclear. Applicability concerns paralleled the use of healthy controls and selected subtypes.

### 3.4 Extracellular chromatin and cell-free DNA biomarkers

Four independent cohorts evaluated histone H3.1, dsDNA, TERT-targeted cfDNA, or beta-globin cfDNA. The bivariate summary sensitivity was 0.712 (95% CI 0.454–0.880) and specificity was 0.796 (0.699–0.868). The corresponding LR + was 3.50 (2.27–5.40), LR − was 0.36 (0.17–0.76), and DOR was 9.68 (3.39–27.62) (Table 3 and Figures 4A, 5A, 6). Sensitivity varied considerably, driven by the low sensitivity of beta-globin cfDNA against hemorrhagic stroke and higher estimates in dsDNA and TERT-cfDNA studies. Only one cohort used stroke mimics, limiting direct applicability to emergency differential diagnosis.

**Table 3:** Primary diagnostic accuracy findings using independent-cohort rules.

Family	*k*	Sensitivity (95% CI)	Specificity (95% CI)	LR+ (95% CI)	LR− (95% CI)	DOR (95% CI)	Interpretation
Extracellular chromatin/cfDNA	4	0.712 (0.454–0.880)	0.796 (0.699–0.868)	3.50 (2.27–5.40)	0.36 (0.17–0.76)	9.68 (3.39–27.62)	Bivariate pooled
Non-coding RNA	10	0.866 (0.829–0.896)	0.922 (0.585–0.990)	11.13 (2.09–87.71)	0.15 (0.11–0.23)	76.6 (9.2–643.3)	Bivariate pooled; wide uncertainty
Hypoxia-response	1	0.647	0.615	1.68	0.57	2.93	HIF-1alpha descriptive only
Protein/multimarker panels	2	0.668–0.920	0.732–0.930	Not pooled	Not pooled	Not pooled	Descriptive range

**Figure 4:**
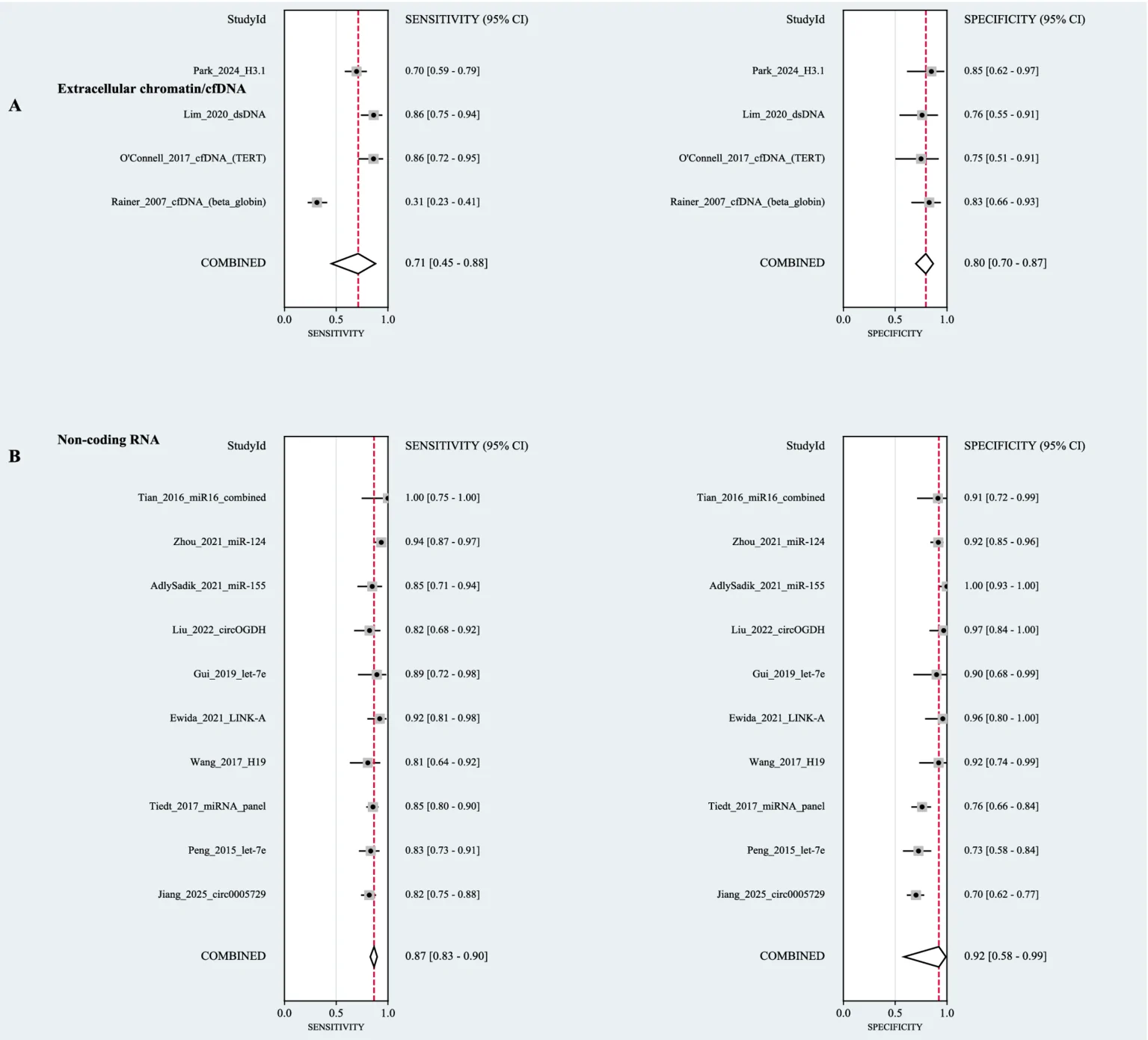
Paired sensitivity and specificity forest plots. **(A)** Extracellular chromatin/cell-free DNA tests. **(B)** Non-coding RNA tests under the one-estimate-per-cohort primary rule. Diamonds show bivariate summary estimates.

**Figure 5:**
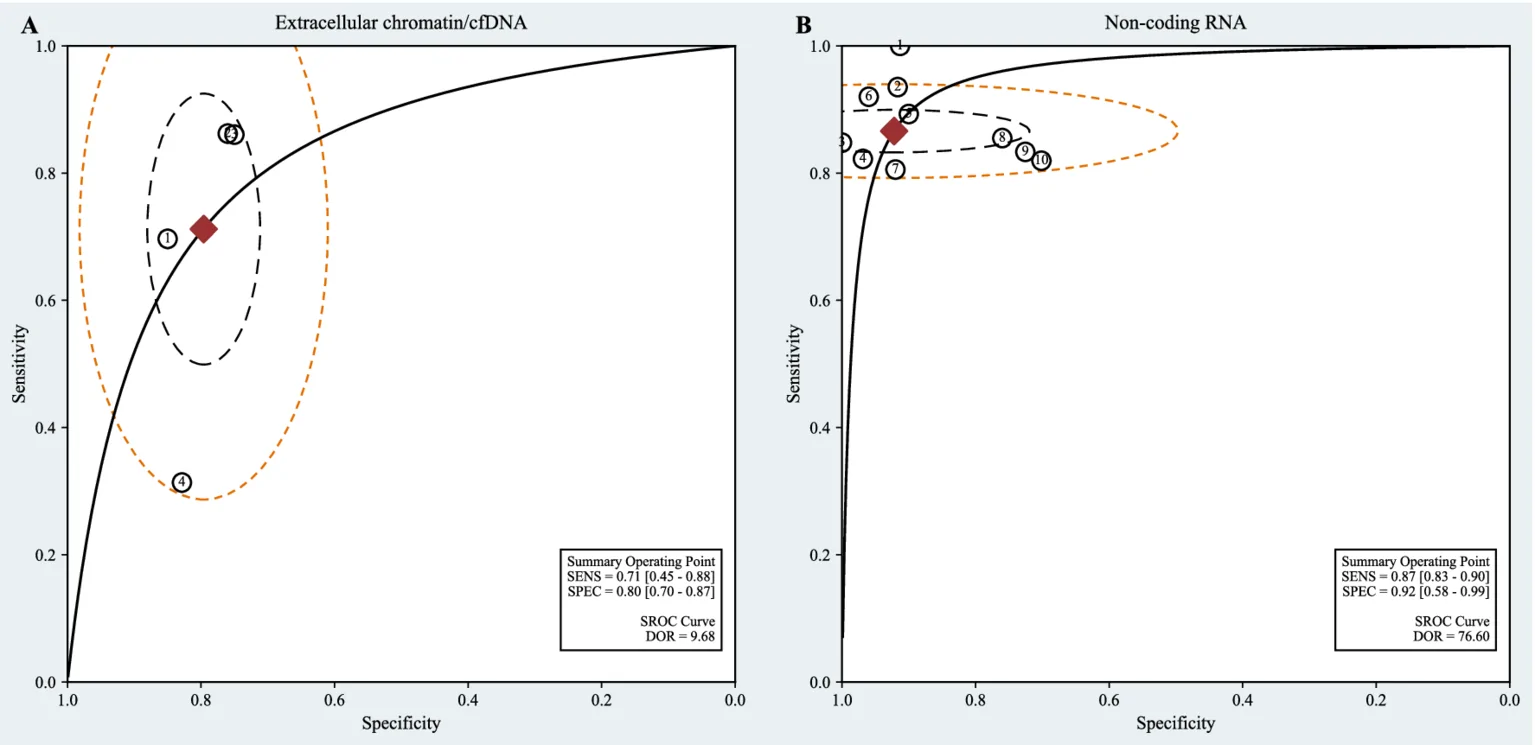
Summary ROC displays. **(A)** Extracellular chromatin/cell-free DNA tests. **(B)** Non-coding RNA tests. Points represent independent cohorts; the summary point and curve should be interpreted in view of comparator-spectrum and threshold heterogeneity.

**Figure 6:**
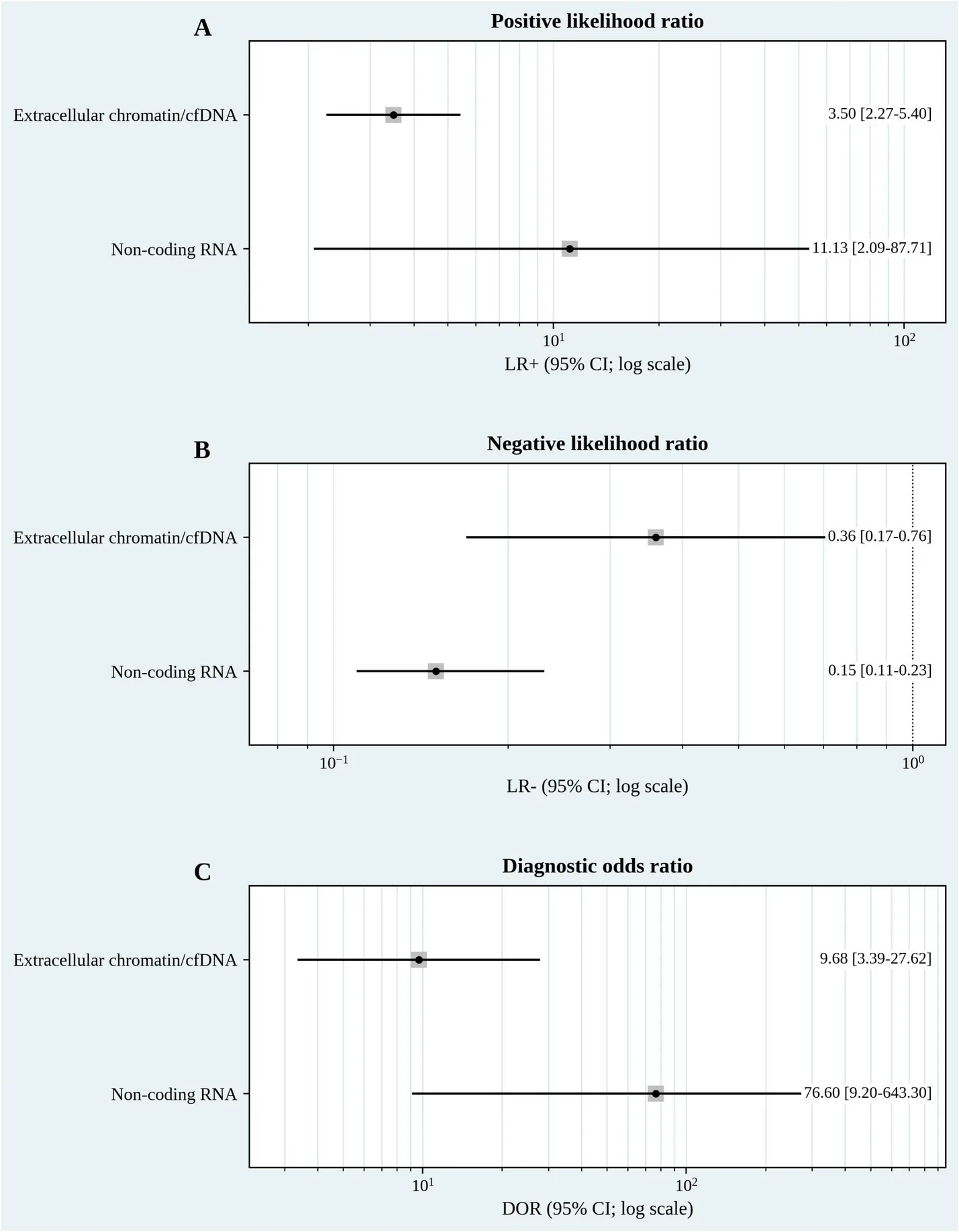
Primary pooled likelihood ratios and diagnostic odds ratios for biomarker families meeting the quantitative pooling threshold. Panels **(A–C)** show LR+, LR−, and DOR, respectively, with 95% confidence intervals. Values correspond to the primary bivariate model reported in Table 3; hypoxia-response and protein/multimarker families were not pooled because fewer than four independent cohorts were available.

### 3.5 Non-coding RNA biomarkers

Ten independent cohorts were retained in the cohort-level non-coding RNA analysis, including the 2025 replication study of hsa_circ_0005729 (29). Pooled sensitivity was 0.866 (95% CI 0.829–0.896) and pooled specificity was 0.922 (0.585–0.990). LR + was 11.13 (2.09–87.71), LR − was 0.15 (0.11–0.23), and DOR was 76.6 (9.2–643.3) (Table 3 and Figures 4B, 5B, 6). Although point estimates were favorable, specificity, LR+, and DOR intervals were very wide, consistent with sparse data, zero cells, and between-study variation. Eight cohorts used healthy controls, so these estimates should not be interpreted as equivalent to performance against stroke mimics (Table 4).

**Table 4:** Comparator spectrum in the cohort-level primary dataset.

Family	Healthy control	Stroke mimic	Hemorrhagic stroke	Risk control	Total
Extracellular chromatin/cfDNA	2	1	1	0	4
Hypoxia-response	1	0	0	0	1
Non-coding RNA	8	0	1	1	10
Protein/multimarker panels	0	2	0	0	2

### 3.6 Hypoxia-response and protein/multimarker tests

The hypoxia-response evidence came from one Rahmati cohort that evaluated both HIF-1alpha and miR-210 (47). HIF-1alpha had sensitivity 0.647 and specificity 0.615 at admission, whereas miR-210 performed less well. Because both tests came from the same 52-case/52-control cohort, no family-level meta-analysis was performed. The protein/multimarker evidence comprised Reynolds et al. (31) and Park et al. (20). The Reynolds five-protein panel was reported to have 92% sensitivity at 93% specificity in early samples, while the Park H-FABP/S100B panel yielded sensitivity 0.668 and specificity 0.732 against stroke mimics. These estimates are descriptive and should not be presented as a pooled protein-family result.

### 3.7 Comparator and sampling-time analyses

Comparator-stratified exploratory summaries are shown in Figure 3 and Supplementary Table S6. Across all biomarker families, healthy-control studies (*k* = 11) yielded sensitivity 0.829 (95% CI 0.773–0.874) and specificity 0.814 (0.736–0.873), whereas stroke-mimic studies (*k* = 3) yielded sensitivity 0.830 (0.615–0.937) and specificity 0.830 (0.608–0.939). Hemorrhagic-stroke comparisons (*k* = 2) were much less precise, with sensitivity 0.689 (0.086–0.981) and specificity 0.892 (0.653–0.973); the single risk-control cohort had sensitivity 0.822 and specificity 0.969. These values should not be interpreted as a direct comparison of biomarker families because the comparator strata contain different assays and study designs. Sampling-window summaries showed sensitivity/specificity of 0.864/0.917 for the hyperacute ≤6 h stratum (*k* = 2), 0.829/0.889 for >6–24 h (*k* = 9), and 0.790/0.705 when exact hours were unclear (*k* = 6) (Supplementary Figure S4 and Supplementary Table S6). Assay-platform meta-regression was not performed because the number of independent cohorts per platform remained insufficient.

### 3.8 Reconstruction and dependence sensitivity analyses

All primary diagnostic cells were reconstructed from published accuracy metrics and group sizes. In the non-coding RNA cohort-level model, floor, nearest-integer, and ceiling reconstruction yielded sensitivities of 0.859, 0.866, and 0.870 and specificities of 0.892, 0.922, and 0.951, respectively (Table 5 and Figure 7). The direction of the result was therefore stable, although LR+ and DOR varied materially because small changes in false-positive counts affect sparse datasets. Leave-one-cohort-out random-effects DOR analyses also showed no single cohort reversed the direction of association: the pooled DOR after omission ranged from 7.60 to 17.00 for extracellular chromatin/cfDNA and from 38.09 to 68.18 for non-coding RNA (Figure 8 and Supplementary Table S7). In a dependence audit, including all eligible non-coding RNA test estimates from shared cohorts reduced the conventional random-effects DOR from 51.31 to 33.81 and modestly reduced pooled sensitivity and specificity (Supplementary Figure S5). Deeks regression for the 10 independent non-coding RNA cohorts did not detect statistically significant asymmetry (*p* = 0.101), although power remained limited (Supplementary Figure S3).

**Table 5:** Rounding-bound sensitivity analysis for the non-coding RNA cohort-level model.

Rule	k	Sensitivity (95% CI)	Specificity (95% CI)	LR+	LR−	DOR
floor	10	0.859 (0.824–0.888)	0.892 (0.796–0.946)	7.98	0.16	50.5
nearest	10	0.866 (0.829–0.896)	0.922 (0.585–0.990)	11.13	0.15	76.6
ceil	10	0.870 (0.836–0.898)	0.951 (0.855–0.984)	17.56	0.14	128.6

**Figure 7:**
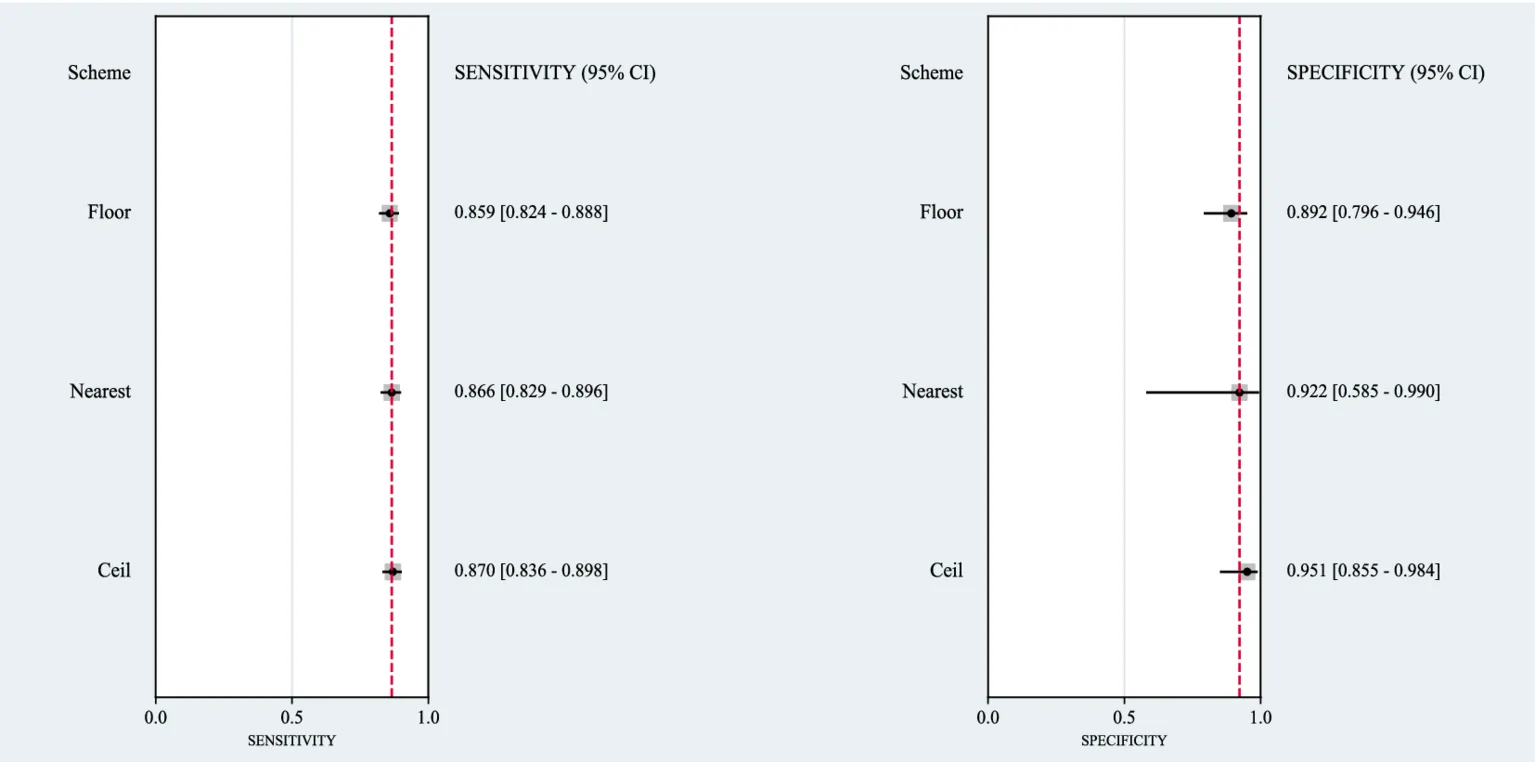
Rounding-bound sensitivity analysis for reconstructed 2 × 2 cells in the non-coding RNA cohort-level model. Floor, nearest-integer, and ceiling reconstructions preserved the direction of pooled sensitivity and specificity but changed ratio measures in sparse cells.

**Figure 8:**
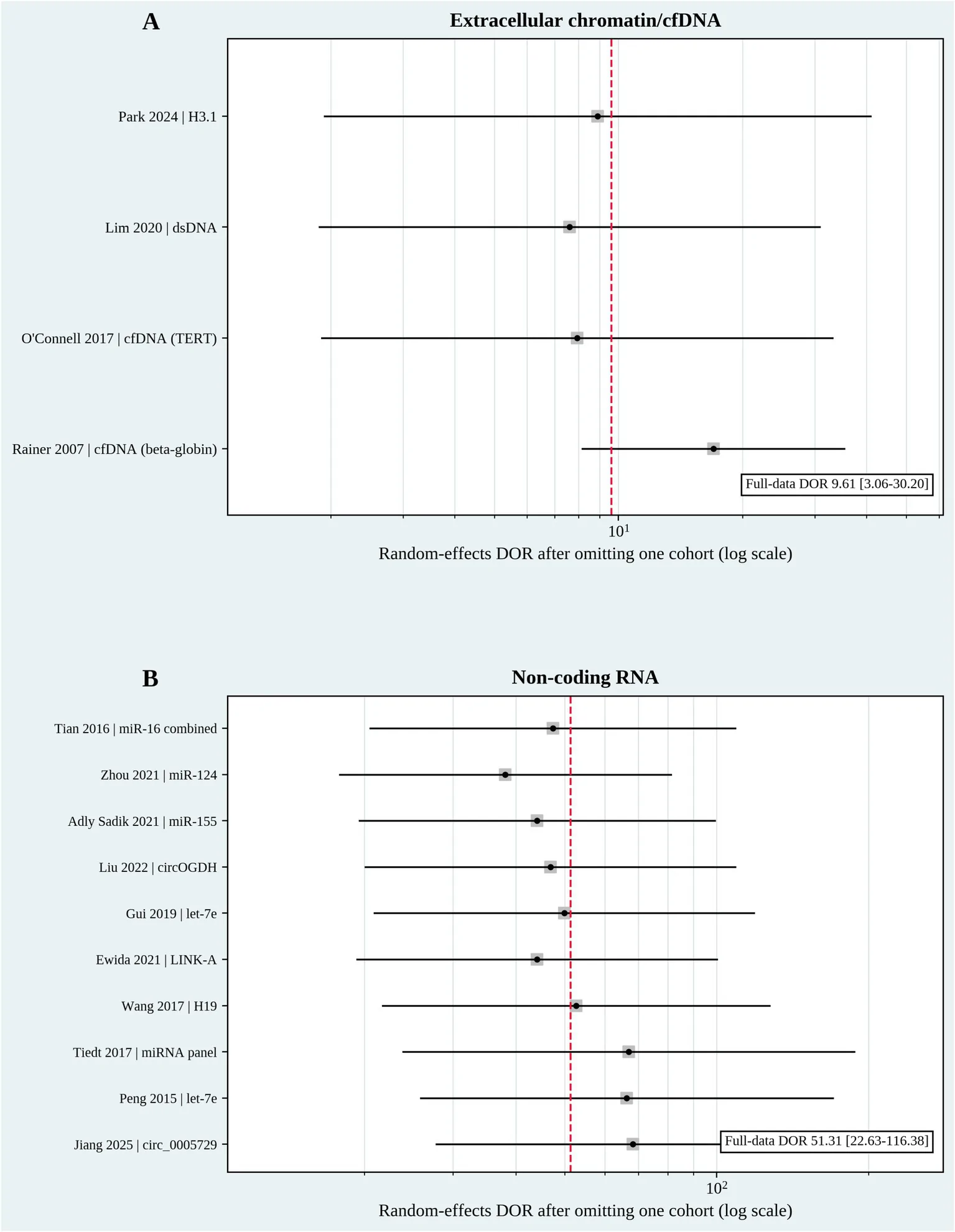
Leave-one-cohort-out influence analysis using random-effects diagnostic odds ratios. Panel **(A)** shows extracellular chromatin/cell-free DNA cohorts and panel **(B)** shows non-coding RNA cohorts. The red dashed line is the full-data DOR under the same conventional random-effects ratio model. This analysis evaluates dominance by individual cohorts and is a sensitivity analysis separate from the primary bivariate diagnostic model.

### 3.9 Supplementary prognostic analysis

The diagnostic analysis was separated from the previously reported prognostic OR synthesis. Four Cit-H3 estimates from one thrombectomy cohort yielded a pooled OR of 0.85 (95% CI 0.56–1.30), whereas a single HIF-1alpha estimate was associated with poor 3-month functional outcome (OR 1.42, 95% CI 1.09–1.83). The overall cross-biomarker estimate was not interpreted because the biomarkers, time points, and constructs differed. These results are presented only in Supplementary Table S5 and Supplementary Figure S2.

## 4 Discussion

### 4.1 Principal findings

This revision materially changes the interpretation of the original manuscript. The strongest numerical diagnostic estimates were observed for non-coding RNA tests, but the evidence was predominantly based on healthy-control, two-gate designs with data-driven thresholds. Extracellular chromatin/cfDNA tests showed moderate pooled discrimination and included the only dedicated cfDNA stroke-mimic cohort. HIF-1alpha and protein/panel evidence was too sparse at the independent-cohort level for valid family pooling. Consequently, the results should be read as exploratory evidence about test families, not a league table of clinical performance.

### 4.2 Non-coding RNA signals and spectrum effects

Circulating miRNAs, lncRNAs, and circRNAs are attractive because they are stable, biologically informative, and measurable early after ischemia (10–14). Primary studies of miR-16, miR-124, miR-155, circOGDH, H19, LINK-A, and multivariable miRNA signatures have reported substantial separation between AIS and controls (29, 33–45, 68). The 2025 PBMC replication study adds contemporary evidence for circRNA-based testing (29). However, high diagnostic performance in selected healthy-control studies can reflect spectrum separation as much as test specificity. Most included RNA cohorts did not enroll consecutive patients with undifferentiated stroke-like symptoms. The wide prediction uncertainty in the cohort-level model and the change in DOR across reconstruction rules reinforce the risk of overinterpretation.

### 4.3 Extracellular chromatin, cfDNA, and NET biology

Extracellular DNA, histones, and related chromatin products have a stronger mechanistic connection to immunothrombosis than the former ‘NETs-related extended’ label implied. NETs are present in retrieved AIS thrombi, can impair fibrinolysis, and are linked to tissue injury and outcome (48–56, 61). Circulating H3.1, dsDNA, and cfDNA therefore have biological plausibility as blood markers (53, 55–60). Yet the pooled diagnostic estimates were only moderate. This is not contradictory: a pathway may be central to thrombus biology without serving as a sufficiently specific emergency diagnostic test. cfDNA is also released by diverse forms of tissue injury, and the Rainer study’s hemorrhagic comparator illustrates how comparator choice changes apparent performance (58).

### 4.4 Hypoxia-response biomarkers

HIF-1alpha is a central regulator of oxygen sensing, metabolic adaptation, angiogenesis, and post-ischemic inflammation (63–65). The single diagnostic cohort suggested only modest discrimination, while other human studies have linked HIF-1alpha more consistently to severity or prognosis (32, 65, 69). The evidence therefore supports HIF-1alpha as a biologically relevant dynamic marker, but not yet as a stand-alone AIS diagnostic test. Treating HIF-1alpha and miR-210 as independent studies would have artificially doubled the evidence from one cohort; the revised analysis avoids that error.

### 4.5 Protein and multimarker panels

Protein panels can integrate neuronal, glial, endothelial, and inflammatory signals. Reynolds et al. reported strong early performance for a five-marker algorithm, whereas the H-FABP/S100B study against stroke mimics showed more modest accuracy (20, 31). The difference is clinically informative: selected panels may appear highly accurate in development samples, but performance often declines in realistic differential-diagnosis cohorts. Recent systematic reviews likewise conclude that multimarker strategies can outperform single biomarkers, but external validation and added-value analyses beyond clinical assessment remain insufficient (8, 9).

### 4.6 Methodological implications

The reviewer-requested checks exposed three major methodological issues. First, “study arms” were not equivalent to independent studies. Multiple markers from Park, Peng, Gui, Rahmati, Tian, and Jiang shared participants. A one-estimate-per-cohort primary analysis is therefore more conservative than the original arm-level synthesis, and the dependence audit confirms that retaining all correlated test estimates changes ratio measures. Second, all 2 × 2 cells were reconstructed; an analysis excluding reconstructed data would contain no studies. Reporting this fact and showing rounding-bound and leave-one-out analyses is preferable to presenting a nominal sensitivity analysis with no valid dataset. Third, comparator heterogeneity is not merely a statistical nuisance. Exploratory stratified summaries produced broadly similar point estimates for healthy-control and stroke-mimic strata, but confidence intervals were wide and heterogeneity remained high; moreover, the strata contained different biomarker families. Healthy controls, hemorrhagic stroke, risk controls, and stroke mimics therefore remain distinct clinical questions and should not be treated as exchangeable (6–9, 22–25, 66, 67).

### 4.7 Clinical relevance

No biomarker family in the present evidence base is ready to replace neuroimaging or structured neurological assessment. Additional cfDNA outcome studies likewise support further validation rather than immediate clinical implementation (59, 60, 70). The most plausible near-term role is adjunctive: accelerating triage, supporting diagnostic confidence where imaging access is delayed, or identifying biologically informative subgroups. A clinically useful rule-in test would require validation in one-gate cohorts of consecutive patients with suspected stroke, including common mimics and intracranial hemorrhage. The current non-coding RNA estimates justify prospective validation, but not routine implementation.

### 4.8 Strengths and limitations

Strengths of the revision include verification of primary reports, resolution of the unidentified protein-panel citation, explicit reclassification of biomarkers, separation of diagnosis from prognosis, cohort-level dependence rules, QUADAS-2 reporting, and transparent reconstruction sensitivity analyses. Limitations remain substantial. The work is a secondary re-analysis rather than a prospectively registered *de novo* review; original database screening counts could not be recovered; all diagnostic cells were reconstructed; several reports used small two-gate designs; thresholds were usually selected in the same datasets; and the number of independent cohorts was insufficient for reliable meta-regression or formal ranking. The QUADAS-2 assessment was performed from published information and may be limited by incomplete reporting.

### 4.9 Conclusion

Circulating non-coding RNA and extracellular chromatin/cfDNA tests show promising diagnostic signals for AIS, but current estimates are vulnerable to selection, threshold, comparator-spectrum, and reconstruction biases. Hypoxia-response and protein/panel families remain underpowered at the independent-cohort level. Future studies should enroll consecutive patients with suspected stroke, prespecify thresholds, standardize sampling windows and assay platforms, report complete 2 × 2 tables, and validate incremental clinical value beyond examination and imaging.

## Data Availability

The original contributions presented in the study are included in the article/Supplementary material, further inquiries can be directed to the corresponding author.
