# Supplementary material for: Diagnostic accuracy of circulating blood biomarker families in acute ischemic stroke: a secondary grouped meta-analysis with primary-study verification
Source: Front Neurol. 2026 Sep 24;17:1856224. doi: 10.3389/fneur.2026.1856224 (PMC13646104; doi:10.3389/fneur.2026.1856224)
Supplement: Supplementary file 1 [file Supplementary_file_1.DOCX]

Supplementary Material

# Supplementary Methods S1. Search strategy and evidence provenance

The revised work is a secondary re-analysis. Candidate reports were identified from Rahmig et al. [8], Zhang et al. [19], backward citation checking, and a targeted PubMed/MEDLINE update through 31 July 2026. The original pre-revision database screening log was not available; therefore, no unrecoverable record counts are reported.

Supplementary Table S1. Search and source-verification strategy.

| **Source** | **Coverage/date** | **Search approach** |
| --- | --- | --- |
| PubMed/MEDLINE | Inception to 31 July 2026 | (("Stroke"[Mesh] OR "Brain Ischemia"[Mesh] OR "acute ischemic stroke"[tiab] OR "ischemic stroke"[tiab] OR "cerebral infarction"[tiab]) AND (biomarker*[tiab] OR blood[tiab] OR serum[tiab] OR plasma[tiab] OR PBMC[tiab]) AND (diagnos*[tiab] OR sensitivity[tiab] OR specificity[tiab] OR ROC[tiab] OR "receiver operating characteristic"[tiab] OR AUC[tiab]) AND ("cell-free DNA"[tiab] OR cfDNA[tiab] OR dsDNA[tiab] OR histone[tiab] OR HIF-1alpha[tiab] OR microRNA*[tiab] OR miRNA*[tiab] OR lncRNA*[tiab] OR circRNA*[tiab] OR H-FABP[tiab] OR S100B[tiab] OR "von Willebrand factor"[tiab] OR MMP-9[tiab])) |
| Rahmig et al. 2024 | Acute-window protein biomarker review | Primary-study tables and backward citation checking |
| Zhang et al. 2025 | Cell-free nucleic-acid review | Primary-study tables and backward citation checking |
| Primary-source verification | Completed 31 July 2026 | DOI/PMID, sample sizes, comparator, threshold, Se/Sp, timing, and assay checked |

# Supplementary Results S2. Complete diagnostic extraction dataset

Supplementary Table S2. Complete verified diagnostic test estimates (24 original estimates).

| **Estimate ID** | **Family** | **Test** | **Comparator** | **AIS/non-AIS** | **TP/FP/FN/TN** | **Se** | **Sp** | **Origin** | **Primary** |
| --- | --- | --- | --- | --- | --- | --- | --- | --- | --- |
| Rainer_2007_beta_globin | Extracellular chromatin/cfDNA | cfDNA (beta-globin) | Hemorrhagic stroke | 118/35 | 37/6/81/29 | 0.310 | 0.830 | Reconstructed from Se/Sp and group sizes | Yes |
| OConnell_2017_cfDNA | Extracellular chromatin/cfDNA | cfDNA (TERT) | Stroke mimic | 43/20 | 37/5/6/15 | 0.860 | 0.750 | Reconstructed from Se/Sp and group sizes | Yes |
| Lim_2020_dsDNA | Extracellular chromatin/cfDNA | dsDNA | Healthy control | 58/25 | 50/6/8/19 | 0.862 | 0.760 | Reconstructed from Se/Sp and group sizes | Yes |
| Park_2024_H3.1 | Extracellular chromatin/cfDNA | Histone H3.1 | Healthy control | 89/20 | 62/3/27/17 | 0.700 | 0.850 | Reconstructed from Se/Sp and group sizes | Yes |
| Rahmati_2021_HIF1a | Hypoxia-response | HIF-1alpha | Healthy control | 52/52 | 34/20/18/32 | 0.647 | 0.615 | Reconstructed from Se/Sp and group sizes | Yes |
| Rahmati_2021_miR210 | Hypoxia-response | miR-210 | Healthy control | 52/52 | 31/18/21/34 | 0.596 | 0.654 | Reconstructed from Se/Sp and group sizes | No |
| Peng_2015_let7e | Non-coding RNA | let-7e | Healthy control | 72/51 | 60/14/12/37 | 0.828 | 0.734 | Reconstructed from Se/Sp and group sizes | Yes |
| Peng_2015_miR338 | Non-coding RNA | miR-338 | Healthy control | 72/51 | 52/24/20/27 | 0.719 | 0.532 | Reconstructed from Se/Sp and group sizes | No |
| Tian_2016_miR16_TACI | Non-coding RNA | miR-16 (TACI) | Healthy control | 4/23 | 4/2/0/21 | 1.000 | 0.913 | Reconstructed from Se/Sp and group sizes | No |
| Tian_2016_miR16_LAA | Non-coding RNA | miR-16 (LAA) | Healthy control | 9/23 | 9/2/0/21 | 1.000 | 0.913 | Reconstructed from Se/Sp and group sizes | No |
| Tiedt_2017_panel | Non-coding RNA | miR-125a-5p/miR-125b-5p/miR-143-3p panel | Healthy control | 200/100 | 171/24/29/76 | 0.856 | 0.763 | Reconstructed from Se/Sp and group sizes | Yes |
| Wang_2017_H19 | Non-coding RNA | lncRNA H19 | Healthy control | 36/25 | 29/2/7/23 | 0.806 | 0.920 | Reconstructed from Se/Sp and group sizes | Yes |
| Gui_2019_let7e | Non-coding RNA | let-7e | Healthy control | 28/20 | 25/2/3/18 | 0.890 | 0.900 | Reconstructed from Se/Sp and group sizes | Yes |
| Gui_2019_miR125b | Non-coding RNA | miR-125b | Healthy control | 28/20 | 24/3/4/17 | 0.860 | 0.870 | Reconstructed from Se/Sp and group sizes | No |
| AdlySadik_2021_miR155 | Non-coding RNA | miR-155 | Healthy control | 46/50 | 39/0/7/50 | 0.857 | 1.000 | Reconstructed from Se/Sp and group sizes | Yes |
| Ewida_2021_LINKA | Non-coding RNA | lncRNA LINK-A | Hemorrhagic stroke | 50/25 | 46/1/4/24 | 0.920 | 0.940 | Reconstructed from Se/Sp and group sizes | Yes |
| Zhou_2021_miR124 | Non-coding RNA | miR-124 | Healthy control | 108/108 | 101/9/7/99 | 0.935 | 0.917 | Reconstructed from Se/Sp and group sizes | Yes |
| Liu_2022_circOGDH | Non-coding RNA | circOGDH | Risk control | 45/32 | 37/1/8/31 | 0.822 | 0.969 | Reconstructed from Se/Sp and group sizes | Yes |
| Jiang_2025_circ0005729 | Non-coding RNA | hsa_circ_0005729 | Healthy control | 144/144 | 118/43/26/101 | 0.822 | 0.701 | Reconstructed from Se/Sp and group sizes | Yes |
| Jiang_2025_circ0075436 | Non-coding RNA | hsa_circ_0075436 | Healthy control | 144/144 | 77/31/67/113 | 0.534 | 0.782 | Reconstructed from Se/Sp and group sizes | No |
| Reynolds_2003_panel | Protein/multimarker panels | vWF/MMP-9/S100B/MCP-1/BNGF panel | Stroke mimic | 82/214 | 75/15/7/199 | 0.920 | 0.930 | Reconstructed from primary-source sensitivity/specificity and reported group sizes | Yes |
| Park_2013_HFABP | Protein/multimarker panels | H-FABP | Stroke mimic | 111/127 | 66/26/45/101 | 0.595 | 0.795 | Reconstructed from Se/Sp and group sizes | No |
| Park_2013_S100B | Protein/multimarker panels | S100B | Stroke mimic | 111/127 | 60/21/51/106 | 0.540 | 0.835 | Reconstructed from Se/Sp and group sizes | No |
| Park_2013_panel | Protein/multimarker panels | H-FABP + S100B panel | Stroke mimic | 111/127 | 74/34/37/93 | 0.668 | 0.732 | Reconstructed from Se/Sp and group sizes | Yes |

# Supplementary Results S3. Primary-source provenance

Supplementary Table S3. Verified primary-source identifiers for included cohorts.

| **Cohort** | **Citation key** | **DOI** | **PMID** | **Primary source** |
| --- | --- | --- | --- | --- |
| Reynolds_2003 | Reynolds 2003 | 10.1373/49.10.1733 | 14500614 | https://pubmed.ncbi.nlm.nih.gov/14500614/ |
| Rainer_2007 | Rainer 2007 | 10.1016/j.cca.2006.08.025 | 17027951 | https://pubmed.ncbi.nlm.nih.gov/17027951/ |
| Park_2013 | Park 2013 | 10.1016/j.clineuro.2012.06.004 | 22766253 | https://pubmed.ncbi.nlm.nih.gov/22766253/ |
| Peng_2015 | Peng 2015 | 10.1007/s12975-015-0422-x | 26415639 | https://pubmed.ncbi.nlm.nih.gov/26415639/ |
| Tian_2016 | Tian 2016 | 10.1371/journal.pone.0166688 | 27846323 | https://pubmed.ncbi.nlm.nih.gov/27846323/ |
| OConnell_2017 | O'Connell 2017 | 10.1080/02699052.2017.1312018 | 28585898 | https://pubmed.ncbi.nlm.nih.gov/28585898/ |
| Tiedt_2017 | Tiedt 2017 | 10.1161/CIRCRESAHA.117.311572 | 28724745 | https://pubmed.ncbi.nlm.nih.gov/28724745/ |
| Wang_2017 | Wang 2017 | 10.14336/AD.2016.0530 | 28203482 | https://pubmed.ncbi.nlm.nih.gov/28203482/ |
| Gui_2019 | Gui 2019 | 10.1007/s12975-018-0659-2 | 30178428 | https://pubmed.ncbi.nlm.nih.gov/30178428/ |
| Lim_2020 | Lim 2020 | 10.1002/jcla.23190 | 31907963 | https://pubmed.ncbi.nlm.nih.gov/31907963/ |
| AdlySadik_2021 | Adly Sadik 2021 | 10.2147/IJGM.S295939 | 33911894 | https://pubmed.ncbi.nlm.nih.gov/33911894/ |
| Ewida_2021 | Ewida 2021 | 10.1007/s12035-021-02440-8 | 34091825 | https://pubmed.ncbi.nlm.nih.gov/34091825/ |
| Rahmati_2021 | Rahmati 2021 | 10.1002/jcla.24073 | 34708885 | https://pubmed.ncbi.nlm.nih.gov/34708885/ |
| Zhou_2021 | Zhou 2021 | 10.1177/10760296211035446 | 34702084 | https://pubmed.ncbi.nlm.nih.gov/34702084/ |
| Liu_2022 | Liu 2022 | 10.1161/CIRCRESAHA.121.319412 | 35189704 | https://pubmed.ncbi.nlm.nih.gov/35189704/ |
| Park_2024 | Park 2024 | 10.1007/s44313-024-00047-1 | 39625632 | https://pubmed.ncbi.nlm.nih.gov/39625632/ |
| Jiang_2025 | Jiang 2025 | 10.3389/fmolb.2025.1657284 |  | https://www.frontiersin.org/journals/molecular-biosciences/articles/10.3389/fmolb.2025.1657284/full |

# Supplementary Results S4. QUADAS-2 judgments

Supplementary Table S4. Study-level QUADAS-2 judgments and rationale.

| **Cohort** | **Patient selection** | **Index test** | **Reference standard** | **Flow/timing** | **Applicability: patients** | **Basis** |
| --- | --- | --- | --- | --- | --- | --- |
| Reynolds_2003 | Unclear | High | Low | Low | Low | Consecutive sampling not fully documented; cutoff selected from study ROC data; AIS confirmed clinically with CT/MRI; acute blood sampling. |
| Rainer_2007 | Unclear | High | Low | Low | Unclear | Consecutive sampling not fully documented; cutoff selected from study ROC data; AIS confirmed clinically with CT/MRI; acute blood sampling. |
| Park_2013 | Unclear | High | Low | Low | Low | Consecutive sampling not fully documented; cutoff selected from study ROC data; AIS confirmed clinically with CT/MRI; acute blood sampling. |
| Peng_2015 | High | High | Low | Unclear | High | Two-gate/selected comparator design; cutoff selected from study ROC data; AIS confirmed clinically with CT/MRI; acute blood sampling. |
| Tian_2016 | High | High | Low | Low | High | Two-gate/selected comparator design; cutoff selected from study ROC data; AIS confirmed clinically with CT/MRI; acute blood sampling. |
| OConnell_2017 | Unclear | High | Low | Low | Low | Consecutive sampling not fully documented; cutoff selected from study ROC data; AIS confirmed clinically with CT/MRI; acute blood sampling. |
| Tiedt_2017 | High | High | Low | Low | High | Two-gate/selected comparator design; cutoff selected from study ROC data; AIS confirmed clinically with CT/MRI; acute blood sampling. |
| Wang_2017 | High | High | Low | Low | High | Two-gate/selected comparator design; cutoff selected from study ROC data; AIS confirmed clinically with CT/MRI; acute blood sampling. |
| Gui_2019 | High | High | Low | Low | High | Two-gate/selected comparator design; cutoff selected from study ROC data; AIS confirmed clinically with CT/MRI; acute blood sampling. |
| Lim_2020 | High | High | Low | Low | High | Two-gate/selected comparator design; cutoff selected from study ROC data; AIS confirmed clinically with CT/MRI; acute blood sampling. |
| AdlySadik_2021 | High | High | Low | Low | High | Two-gate/selected comparator design; cutoff selected from study ROC data; AIS confirmed clinically with CT/MRI; acute blood sampling. |
| Ewida_2021 | Unclear | High | Low | Low | Unclear | Consecutive sampling not fully documented; cutoff selected from study ROC data; AIS confirmed clinically with CT/MRI; acute blood sampling. |
| Rahmati_2021 | High | High | Low | Low | High | Two-gate/selected comparator design; cutoff selected from study ROC data; AIS confirmed clinically with CT/MRI; acute blood sampling. |
| Zhou_2021 | High | High | Low | Low | High | Two-gate/selected comparator design; cutoff selected from study ROC data; AIS confirmed clinically with CT/MRI; acute blood sampling. |
| Liu_2022 | High | High | Low | Low | High | Two-gate/selected comparator design; cutoff selected from study ROC data; AIS confirmed clinically with CT/MRI; acute blood sampling. |
| Park_2024 | High | High | Low | Low | High | Two-gate/selected comparator design; cutoff selected from study ROC data; AIS confirmed clinically with CT/MRI; acute blood sampling. |
| Jiang_2025 | High | High | Low | Low | High | Two-gate/selected comparator design; cutoff selected from study ROC data; AIS confirmed clinically with CT/MRI; acute blood sampling. |

# Supplementary Results S5. Additional analyses

Supplementary Table S5. Prognostic and audit sensitivity analyses.

| **Analysis** | **k** | **Estimate** | **95% CI** | **Interpretation** |
| --- | --- | --- | --- | --- |
| Cit-H3 prognostic OR | 4 test/time-point estimates from one cohort | 0.85 | 0.56-1.30 | No clear association; estimates are not independent studies |
| HIF-1alpha prognostic OR | 1 | 1.42 | 1.09-1.83 | Single-study adverse association |
| Original arm-level non-coding RNA metandi model | 10 arms | Se 0.879; Sp 0.922; DOR 85.78 | Se 0.846-0.905; Sp 0.866-0.956; DOR 43.46-169.33 | Audit result; narrower intervals partly reflect correlated tests |
| Cohort-level non-coding RNA model | 10 cohorts | Se 0.866; Sp 0.922; DOR 76.6 | Se 0.829-0.896; Sp 0.585-0.990; DOR 9.2-643.3 | Primary conservative analysis |

# Supplementary Figures

Supplementary Figure S1. Study-level QUADAS-2 traffic-light plot across 17 verified primary reports. Green (+), low risk; yellow (?), unclear risk; red (−), high risk.

Supplementary Figure S2. Separate prognostic random-effects forest plot for Cit-H3 and HIF-1alpha odds-ratio estimates. The four Cit-H3 estimates arise from one thrombectomy cohort at different treatment/time-point conditions and are not independent studies. The HIF-1alpha estimate is a single study. This analysis is not part of the diagnostic accuracy synthesis.

Supplementary Figure S3. Exploratory Deeks regression plot for the 10 independent non-coding RNA cohorts. The weighted regression slope was not statistically significant (p=0.101). Because only 10 cohorts were available and study designs were heterogeneous, absence of statistical asymmetry should not be interpreted as evidence that publication bias is absent.

Supplementary Figure S4. Exploratory sampling-window subgroup summaries. Sensitivity and specificity were summarized separately with univariate random-effects logit models. The exact-hour-unclear stratum includes studies reported only as admission, presentation, emergency admission, or acute stage; these estimates are descriptive and do not replace the primary family-specific bivariate models.

Supplementary Figure S5. Dependence sensitivity analysis for non-coding RNA estimates. One-estimate-per-cohort results are compared with an analysis retaining all eligible test estimates from shared cohorts using the same conventional random-effects summaries. The analysis illustrates the effect of within-cohort multiplicity and is not a substitute for the primary bivariate model.

# Supplementary Tables S6-S7. Reviewer-requested heterogeneity and influence analyses

Supplementary Table S6. Exploratory comparator-type and sampling-window summaries. These univariate random-effects summaries are descriptive and do not replace the primary bivariate family-specific models.

| **Stratifier** | **Stratum** | **k** | **Sensitivity (95% CI)** | **Specificity (95% CI)** | **Interpretation** |
| --- | --- | --- | --- | --- | --- |
| Comparator | Healthy control | 11 | 0.829 (0.773-0.874) | 0.814 (0.736-0.873) | Exploratory; biomarker families differ across comparator strata |
| Comparator | Stroke mimic | 3 | 0.830 (0.615-0.937) | 0.830 (0.608-0.939) | Exploratory; biomarker families differ across comparator strata |
| Comparator | Hemorrhagic stroke | 2 | 0.689 (0.086-0.981) | 0.892 (0.653-0.973) | Exploratory; biomarker families differ across comparator strata |
| Comparator | Risk control | 1 | 0.822 (0.683-0.909) | 0.969 (0.809-0.996) | Exploratory; biomarker families differ across comparator strata |
| Sampling window | Hyperacute (≤6 h) | 2 | 0.864 (0.576-0.968) | 0.917 (0.798-0.968) | Exploratory; exact sampling definitions varied across studies |
| Sampling window | Early acute (>6–24 h) | 9 | 0.829 (0.674-0.919) | 0.889 (0.811-0.937) | Exploratory; exact sampling definitions varied across studies |
| Sampling window | Admission/presentation; exact hours unclear | 6 | 0.790 (0.715-0.849) | 0.705 (0.651-0.753) | Exploratory; exact sampling definitions varied across studies |

Supplementary Table S7. Leave-one-cohort-out DOR influence analysis.

| **Family** | **Omitted cohort/test** | **Remaining k** | **DOR** | **95% CI** |
| --- | --- | --- | --- | --- |
| Extracellular chromatin/cfDNA | Park 2024 \| H3.1 | 3 | 8.90 | 1.93-40.95 |
| Extracellular chromatin/cfDNA | Lim 2020 \| dsDNA | 3 | 7.60 | 1.88-30.81 |
| Extracellular chromatin/cfDNA | O'Connell 2017 \| cfDNA (TERT) | 3 | 7.93 | 1.90-33.07 |
| Extracellular chromatin/cfDNA | Rainer 2007 \| cfDNA (beta-globin) | 3 | 17.00 | 8.17-35.36 |
| Non-coding RNA | Tian 2016 \| miR-16 combined | 9 | 47.32 | 20.56-108.90 |
| Non-coding RNA | Zhou 2021 \| miR-124 | 9 | 38.09 | 17.88-81.13 |
| Non-coding RNA | Adly Sadik 2021 \| miR-155 | 9 | 44.07 | 19.58-99.16 |
| Non-coding RNA | Liu 2022 \| circOGDH | 9 | 46.82 | 20.12-108.96 |
| Non-coding RNA | Gui 2019 \| let-7e | 9 | 49.86 | 20.97-118.58 |
| Non-coding RNA | Ewida 2021 \| LINK-A | 9 | 44.03 | 19.37-100.09 |
| Non-coding RNA | Wang 2017 \| H19 | 9 | 52.64 | 21.76-127.36 |
| Non-coding RNA | Tiedt 2017 \| miRNA panel | 9 | 66.96 | 23.90-187.57 |
| Non-coding RNA | Peng 2015 \| let-7e | 9 | 66.34 | 25.88-170.08 |
| Non-coding RNA | Jiang 2025 \| circ_0005729 | 9 | 68.18 | 27.79-167.27 |
